# Adaptive Athlete Management in Youth Football: A Developmentally Informed Framework for Applied Practice

**DOI:** 10.3390/sports14080324

**Published:** 2026-08-01

**Authors:** Nikolaos Androulakis, Nikolaos Koundourakis

**Affiliations:** 1 Hematology Laboratory, University Hospital of Ιraklion, 71500 Ιraklion, Crete, Greece; 2 Performance Department, O.F.I. Soccer Club, 71500 Ιraklion, Crete, Greece; nkoundourakis@mitropolitiko.edu.gr

**Keywords:** youth football, athlete monitoring, biological maturation, bio-banding, long-term athlete development

## Abstract

Youth football academies increasingly utilize physical testing, athlete monitoring, anthropometric profiling, and performance analysis to support athlete development and training management. During adolescence, rapid biological maturation, fluctuating neuromuscular coordination, variable recovery capacity, and ongoing psychological development may substantially influence physical and football-specific performance over time. Consequently, athlete-management decisions based exclusively on isolated testing outcomes may inadequately reflect the developmental needs of young players. This narrative review presents a developmentally informed framework for applied athlete management in youth football academies. The review discusses the interaction between football-specific technical and tactical qualities, physical performance characteristics, biological maturation, somatometric profile, psychological and perceptual-cognitive factors, recovery management, and monitoring strategies during adolescent development. Particular emphasis is placed on practical implementation within real-world academy environments, including low-cost monitoring approaches, intra-club bio-banding, multidisciplinary communication, and athlete-specific intervention planning. Overall, the proposed framework positions athlete monitoring as a continuous support process designed to guide training adjustment, recovery management, and sustainable football progression within youth football development pathways.

## 1. Introduction

Modern youth football increasingly incorporates physical testing, athlete monitoring, performance analysis, and developmental profiling into everyday academy practice. Sprint assessment, jump testing, anthropometric evaluation, wellness monitoring, recovery tracking, and strength assessment are now commonly utilized across multiple competitive levels to support athlete development and training management [1,2]. Advances in sports science, monitoring technologies, and field-based testing methodologies have further expanded the availability of objective performance data within youth football settings [3,4].

Within the context of the present review, real-world academy environments refer to organized youth football structures possessing access to basic field-testing equipment and routine monitoring practices, yet frequently operating without fully integrated multidisciplinary performance departments. In such settings, practical challenges often relate less to the absence of technology and more to limitations in staffing, time availability, data interpretation, and coach workload. Recent evidence from a professional academy environment similarly indicates that staffing limitations, time constraints, stakeholder communication, and the need to translate complex outputs into meaningful information may restrict the effective implementation of performance-analysis technologies [5]. Coaches are therefore frequently required to simultaneously manage training organization, technical instruction, monitoring procedures, and athlete-management decisions within highly time-constrained developmental environments.

Despite the increasing availability of monitoring data, athlete evaluation in youth football frequently remains influenced by approaches in which isolated physical or anthropometric characteristics are disproportionately emphasized during talent identification and developmental decision-making [6,7]. Sprint performance, jump capacity, body composition, or temporary maturation-related physical advantages may sometimes be interpreted as direct indicators of long-term football potential despite the multifactorial nature of football performance itself. Such tendencies may underestimate the contribution of technical, tactical, perceptual-cognitive, psychological, and developmental factors that substantially influence long-term progression and competitive effectiveness.

These challenges become particularly relevant during adolescence. Rapid growth, biological maturation, fluctuating neuromuscular coordination, psychological development, and variable training responses may substantially alter performance characteristics over relatively short periods of time [8,9]. During these phases, temporary physical advantages or disadvantages may emerge independently of long-term football potential, increasing the risk of premature classification or exclusion within academy systems. Contemporary long-term athlete-development approaches therefore emphasize individualized progression, developmental timing, and the interaction between biological, psychological, technical, social, and environmental influences during adolescent development [9,10,11].

Despite the increasing implementation of athlete-monitoring systems within youth football, practical models guiding how monitoring information should support day-to-day athlete-management decisions remain limited [12]. Contemporary studies of academy practice indicate that practitioners value multidimensional monitoring but may also encounter uncertainty regarding how particular methods should be implemented, interpreted, communicated, and integrated into routine developmental programmes [5,13]. In applied academy environments, effective athlete development may therefore benefit from the integration of football-specific coaching, physical assessment, maturation monitoring, recovery management, and psychosocial support within a feasible longitudinal management process [14].

Established frameworks -including long-term athlete-development approaches, the Youth Physical Development Model, the Athletic Skills Model, and holistic ecological perspectives- have substantially advanced understanding of athlete development by emphasizing long-term progression, multidimensional development, environmental influences, and the interaction between individual and contextual constraints [9,10,11,15,16]. However, these frameworks were not primarily developed to explain how football-specific observations, physical assessments, maturation estimates, psychological information, and recovery-related monitoring should be combined to inform everyday athlete-management decisions within resource-constrained academy environments. Similarly, much of the athlete-monitoring literature focuses on the validity, reliability, or interpretation of individual tests and technologies rather than on the operational process through which heterogeneous information is converted into individualized developmental decisions [4,5].

Rather than proposing an alternative to existing developmental models, the present review seeks to address the implementation gap between multidimensional athlete assessment and everyday decision-making in youth football academies. Specifically, it proposes a practical conceptual framework that integrates football-specific observations, physical assessment, biological maturation, recovery, and psychosocial information into a continuous process of contextual interpretation, individualized decision-making, targeted intervention, and reassessment. The framework is intended as a conceptually informed decision-support approach rather than a validated athlete-management system, and its practical effectiveness requires prospective longitudinal evaluation.

Accordingly, the aim of this narrative review was to synthesize current evidence across the principal domains of youth football athlete development and to translate this evidence into a practical, developmentally informed framework for adaptive athlete management. Rather than focusing on the validity of individual assessment methods, the review emphasizes how multidimensional information can be integrated to support individualized coaching, training modification, and longitudinal athlete-management decisions within everyday academy practice.

### Literature Search Strategy

This narrative review was informed by a structured literature search covering youth football development, biological maturation, athlete monitoring, physical performance assessment, recovery and training-load management, psychological and perceptual-cognitive development, and long-term athlete development. PubMed, Scopus, and SPORTDiscus were searched for English-language literature published from January 2000 to January 2026. Earlier publications were retained when they represented foundational contributions to the concepts examined. Search terms were combined as appropriate and included “youth football”, “soccer”, “athlete monitoring”, “biological maturation”, “bio-banding”, “peak height velocity”, “physical performance”, “training load”, “recovery”, “talent identification”, “psychological development”, “ecological dynamics”, and “long-term athlete development”. Reference lists of relevant studies, reviews, consensus statements, and position papers were also examined to identify additional literature.

Peer-reviewed original investigations, longitudinal studies, systematic and narrative reviews, meta-analyses, consensus statements, position papers, and relevant scholarly books were considered. Publications were included when they contributed directly to one or more domains of the proposed framework or addressed the implementation and interpretation of athlete-management practices in adolescent or football-academy settings. Non-peer-reviewed reports, conference abstracts, unsupported opinion articles, and publications without direct relevance to the review objectives were excluded. Titles and abstracts were screened for relevance, followed by full-text consideration of potentially eligible publications.

Because the review sought to integrate evidence from several interconnected domains rather than answer a single narrowly defined question or estimate a pooled effect, a narrative methodology and thematic synthesis were considered appropriate. Evidence was organized according to the principal domains of adaptive athlete management and interpreted with emphasis on developmental context and practical applicability. Where empirical support was limited or indirect, statements are presented as cautious, conceptually informed, or practice-based recommendations requiring future validation.

## 2. Football-Specific Talent as the Core of Athlete Evaluation

### Technical, Tactical, and Game-Related Characteristics

Football performance in youth academies cannot be adequately explained through physical capacities alone. Although sprint ability, strength, power, and anthropometric characteristics may substantially influence match performance, football remains a highly interactive and non-linear sport in which technical execution, tactical understanding, perceptual awareness, decision-making, and adaptability strongly influence competitive effectiveness [7,17,18,19]. Athlete-development models should therefore prioritize football-specific qualities when evaluating player progression during adolescent development.

Technical and tactical competencies frequently differentiate successful developmental trajectories, even when athletes present less favorable physiological profiles during standardized testing [17]. Ball control, spatial occupation, exploratory visual scanning behavior, timing of movement, and tactical adaptation under pressure may substantially influence match contribution independently of maximal physical output [17,20]. In applied academy settings, coaches often observe athletes who consistently demonstrate high-level game effectiveness despite presenting lower sprint, strength, or anthropometric values during field testing. Such observations reinforce the importance of evaluating football-specific functionality alongside physical-assessment results.

Importantly, comparable football-specific outcomes may emerge through different developmental processes. For example, an athlete may dominate 1v1 duels at the U13 level primarily due to temporary maturation-related physical advantages, whereas comparable success at the U16 level may depend more heavily on advanced neuromuscular coordination, perceptual timing, and tactical adaptation. Coaches should therefore consider maturational status, training history, and developmental stage when evaluating football-specific performance.

Beyond technical execution, perceptual-cognitive characteristics represent a major component of football expertise. The ability to rapidly interpret environmental information, anticipate evolving situations, recognize tactical patterns, and adapt decisions under pressure substantially influences performance behavior during match play [21,22,23]. However, these qualities are often more difficult to quantify reliably compared with physical assessments, increasing the importance of structured observational analysis and repeated observation within academy environments.

In real-world academy settings lacking advanced technological infrastructure, practical observational tools such as the Game Performance Assessment Instrument (GPAI) may assist coaches in organizing football-specific evaluation during small-sided games and training situations. More recently, low-cost video-analysis approaches and wearable tracking systems have further expanded the accessibility of applied tactical and movement assessment within academy environments [4]. Furthermore, representative learning environments such as appropriately designed small-sided games may provide ecologically valid opportunities to simultaneously evaluate technical execution, tactical behaviour, perceptual-cognitive performance, and decision-making under representative game constraints [18,19,24]. Nevertheless, observational tools should primarily support ongoing coaching decisions rather than function as rigid grading systems. Repeated observation across different developmental phases may therefore provide greater practical insight than isolated single-time-point assessment, particularly during periods of rapid growth, tactical adaptation, or fluctuating performance characteristics.

Football-specific qualities should additionally be considered alongside positional role, tactical system, competitive level, and maturational stage. The performance demands imposed on wide attacking players differ considerably from those required of central midfielders, central defenders, or forwards, resulting in distinct technical, tactical, and physical priorities across positions. Wide players may rely more heavily on repeated acceleration exposure and open-space actions, whereas central midfielders may depend more extensively on spatial awareness, tempo regulation, exploratory scanning behavior, and continuous decision-making involvement. Standardized evaluation models may therefore inadequately capture the positional and developmental variability observed within youth football environments.

Physical testing, maturation monitoring, recovery assessment, and somatometric profiling may provide valuable complementary information; however, their usefulness depends largely on how effectively they inform coaching decisions, training adjustment, and developmental planning within the demands of everyday academy practice.

## 3. Physical Performance Assessment

### Sprint, Acceleration, Jumping Ability, and Strength

Physical performance characteristics represent important components of athlete evaluation due to the demanding nature of contemporary match play. Repeated high-intensity actions, rapid transitions, accelerations, decelerations, and duel situations frequently influence competitive outcomes across multiple playing positions [1,25]. The assessment of sprint capacity, jump ability, and strength characteristics has therefore become increasingly integrated into youth football academies. However, the practical usefulness of these metrics depends not only on measurement itself, but also on testing reliability, appropriate interpretation, and longitudinal monitoring [3,4].

Sprint and acceleration capacities are particularly relevant given the intermittent and transition-oriented nature of football. Although maximal sprint velocity contributes to certain match situations, short-distance acceleration ability may be more directly associated with pressing actions, space creation, defensive recovery, directional transitions, and first-step responsiveness [26,27]. Acceleration performance should therefore be evaluated alongside the athlete’s maturational status, training exposure, movement competency, positional demands, and recovery condition. Temporary reductions in sprint performance may reflect multiple interacting factors, including fatigue accumulation, rapid growth, altered coordination, insufficient force-production capacity, or technical inefficiency.

The methodological quality of physical testing procedures additionally represents an important practical consideration within academy environments. Manual stopwatch timing, despite its continued use in some developmental settings, may introduce substantial measurement error due to reaction-time variability and inconsistent timing initiation, particularly during short sprint assessments where small performance differences may possess practical importance [28]. Electronic timing gates may therefore substantially improve measurement precision and testing reliability during sprint and acceleration assessment.

In academy environments where advanced laboratory infrastructure is unavailable, validated low-cost technological solutions may provide useful alternatives. Applications such as MyJump have demonstrated high agreement and excellent reliability compared with force-platform methodologies for jump assessment, supporting their practical implementation within real-world academy settings [29,30,31]. Additionally, video-analysis and movement-screening applications may provide useful complementary information regarding movement mechanics, postural asymmetries, mobility limitations, and coordination changes during adolescence. Although such approaches should not replace formal biomechanical assessment, they may assist practitioners in identifying potentially relevant movement alterations within resource-limited academy settings, particularly during periods of rapid growth and fluctuating neuromuscular coordination [32].

Recent advances in wearable sensor technology, inertial measurement systems, and field-based tracking platforms have further expanded the accessibility of athlete monitoring within applied sport environments [33]. Small performance fluctuations should additionally be interpreted cautiously, particularly when changes remain within the typical error of the measurement procedure. Technological sophistication ultimately remains secondary to methodological consistency and practical implementation within everyday academy practice.

Jump-based assessments, particularly the countermovement jump (CMJ) and squat jump (SJ), are widely utilized in youth football due to their practical feasibility and their association with lower-limb neuromuscular performance. These assessments may provide useful information regarding explosive force production, stretch-shortening cycle utilization, training adaptation, and neuromuscular fatigue status [34,35]. Longitudinal changes in jump performance may additionally assist practitioners in monitoring adaptation patterns and recovery responses throughout the competitive season. Nevertheless, jump-derived outcomes should be interpreted within their biological, methodological, and training context, because similar reductions may reflect accumulated fatigue, maturational change, altered movement strategy, or normal intra-individual variability rather than impaired readiness alone [4,35]. Coaches should therefore interpret jump-performance data alongside the athlete’s current training load, recovery condition, and developmental stage.

Lower-limb force-production characteristics may substantially influence acceleration mechanics, deceleration control, movement efficiency, physical robustness, and repeated high-intensity football performance [36,37]. These factors become particularly relevant during periods of rapid growth, where transient coordination disturbances (“adolescent awkwardness”), altered movement mechanics, and reduced neuromuscular control may increase vulnerability to overload conditions and repetitive stress injuries. Insufficient movement competency, reduced tissue tolerance, and limited mobility or elasticity may further contribute to mechanical inefficiency and increased injury susceptibility if not appropriately addressed through age and maturation-appropriate strength and movement interventions. Nevertheless, force-production characteristics in youth athletes should always be interpreted within the athlete’s current maturational and developmental status rather than chronological age alone.

Physical assessment should ultimately support practical athlete-management decisions rather than function as an isolated grading process. Longitudinal monitoring of sprint, jump, and strength characteristics may assist practitioners in identifying emerging limitations, guiding individualized training adjustments, and monitoring developmental adaptation within youth football environments. However, the practical value of physical-performance testing depends on its integration with football-specific observations, maturational status, training-load information, recovery condition, and the broader developmental context [4].

## 4. Growth, Biological Maturation, and Somatometric Characteristics

### Developmental Variability and Contextual Interpretation in Youth Football

Growth and biological maturation represent fundamental components of athlete evaluation in youth football. Unlike adult populations, youth athletes experience ongoing developmental shifts that may substantially alter sprint capacity, strength characteristics, movement efficiency, coordination, and fatigue tolerance over relatively short periods of time [38,39]. Maturational status should therefore be routinely considered when evaluating physical performance, training responses, and football-specific development during adolescence.

A central challenge in youth academies involves distinguishing temporary maturation-related advantages from genuine long-term football potential. Early-maturing athletes frequently demonstrate superior height, body mass, acceleration, and power characteristics during pubertal development compared with later-maturing peers, which may contribute to preferential selection and increased competitive exposure within academy systems [38,40]. These tendencies may be further amplified by the Relative Age Effect (RAE), whereby athletes born earlier within the selection year experience developmental and competitive advantages during youth progression pathways [41]. Conversely, late-maturing players may initially appear physically disadvantaged despite possessing highly developed technical, perceptual-cognitive, or psychological qualities that become increasingly influential during later developmental stages.

To reduce these biases, academies have increasingly explored strategies such as bio-banding, whereby athletes are grouped according to biological maturation rather than chronological age alone [42,43]. Bio-banding has been proposed as an approach that may reduce maturational selection bias and facilitate developmentally appropriate training and competition; however, current evidence regarding its long-term effects on talent identification, athlete development, and progression remains limited [44]. Beyond reducing selection bias, bio-banding may additionally assist coaches in modifying tactical demands, physical exposure, and developmental challenges according to the athlete’s current maturational stage. Early-maturing athletes may therefore be exposed to greater tactical and decision-making demands, whereas later-maturing players may receive increased opportunity to express and refine technical qualities within more developmentally appropriate competitive environments.

Somatometric characteristics may further influence football performance throughout development. Stature, limb length, lean body mass, and body composition may affect acceleration mechanics, change-of-direction efficiency, repeated high-intensity performance, movement economy, and physical robustness during duel situations [45,46]. However, structural characteristics should not be viewed as fixed determinants of long-term football success. Positional role, tactical system, movement strategy, technical proficiency, and maturational timing may substantially modify how physical characteristics influence practical match performance within academy environments.

These interactions become particularly important during periods of accelerated pubertal growth. Rapid skeletal development and changing body proportions frequently disrupt neuromuscular coordination, movement control, and mechanical efficiency, resulting in transient coordination disturbances commonly referred to as “adolescent awkwardness” [39]. Sudden reductions in movement efficiency, sprint performance, or coordination during Peak Height Velocity (PHV) should therefore not automatically be interpreted as reductions in talent or long-term potential, but rather as possible biological warning signs requiring careful reassessment. Altered force absorption, fluctuating movement mechanics, and reduced neuromuscular control during this developmental phase may increase susceptibility to growth-related overload conditions and repetitive stress injuries, including traction apophyseal disorders such as Osgood–Schlatter and Sever’s disease, as well as stress-related conditions including osteitis pubis and lumbar spondylolysis [47].

Appropriate intervention strategies may therefore involve temporary load modification, mobility support, movement retraining, early lumbopelvic stabilization, and maturation-appropriate strength interventions rather than excessive corrective technical training.

From an applied perspective, academies lacking advanced laboratory infrastructure may still effectively monitor maturation using practical non-invasive approaches such as the Mirwald equations (maturity offset) or the Khamis–Roche method (predicted adult stature) [48,49]. However, these methods should be interpreted cautiously within academy environments. Evidence has highlighted important limitations of maturity-offset prediction equations, including reduced accuracy at the individual level, systematic bias toward the group mean, and decreased precision in early- and late-maturing athletes [50,51]. Additional methodological concerns have also been raised regarding the practical interpretation of predicted age at Peak Height Velocity and the risk of misclassification when maturity-offset equations are used as definitive maturational markers rather than contextual estimation tools [50,51]. Prediction accuracy may further vary according to maturation timing, anthropometric measurement consistency, and individual variability, while inaccurate standing-height or sitting-height measurements may substantially influence maturity-offset estimations. Similarly, Khamis–Roche predictions may be affected by parental-height reporting inaccuracies when self-reported values are used instead of directly measured anthropometric data.

Longitudinal monitoring of biological development may assist practitioners in adjusting training loads, contextualizing temporary performance fluctuations, reducing premature talent classification, and guiding developmentally appropriate training decisions within youth football environments. However, maturation-related information should always be interpreted alongside football-specific performance, technical and tactical development, and the athlete’s broader developmental context rather than as an isolated determinant of current or future potential.

## 5. Psychological, Behavioral, and Perceptual-Cognitive Characteristics

### Continuously Evolving Developmental States

Psychological, behavioral, and perceptual-cognitive characteristics represent important but highly variable components of athlete evaluation in youth football. Unlike many physical-performance variables, these characteristics are difficult to quantify objectively and may fluctuate considerably due to educational, competitive, social, and environmental influences. Decision-making, adaptability, emotional regulation, learning responsiveness, motivation, and game awareness may all substantially influence football-specific performance and long-term development within academy environments [23,52]. Psychological and behavioral characteristics should therefore be viewed as dynamic developmental factors rather than fixed personal traits.

The ability to process dynamic game information under time pressure represents a major component of football expertise. Anticipation, pattern recognition, exploratory visual scanning behavior, situational awareness, and rapid decision-making strongly influence tactical effectiveness and technical execution during match play [21,23,53]. These perceptual-cognitive abilities may additionally allow some athletes to compensate for temporary physical limitations through improved positioning, timing, and movement efficiency. However, such abilities are also influenced by tactical systems, coaching philosophy, competitive demands, accumulated playing experience, mental fatigue, emotional stress, and academic burden outside football. Repeated observational assessment may therefore provide greater practical insight than isolated single-time-point evaluation.

Mental fatigue represents an increasingly recognized component of athlete monitoring in youth football and should be considered separately from physical fatigue. Academic workload, prolonged cognitive effort, congested competition schedules, psychosocial stressors, travel demands, and accumulated decision-making requirements may all contribute to transient mental fatigue during adolescence [54,55]. Recent evidence further suggests that mental fatigue may impair decision-making, technical execution, vigilance, reaction time, perceptual-cognitive performance, and tactical behaviour, while exploratory findings also indicate potential associations with external-load characteristics derived from GPS monitoring [56]. Consequently, temporary reductions in football-specific performance should not automatically be interpreted as diminished motivation, reduced talent, or inadequate training adaptation, but rather assessed within the athlete’s broader cognitive, psychological, educational, and recovery context.

Behavioral characteristics such as motivation, coachability, resilience, emotional control, training consistency, and recovery adherence may substantially influence the athlete’s ability to tolerate training demands and adapt positively to competitive challenges [57,58]. Their evaluation becomes particularly challenging during adolescence, where emotional maturation, peer relationships, academic stress, social influences, and competitive pressure may strongly affect behavioral consistency. Temporary fluctuations in confidence, engagement, emotional stability, or responsiveness to coaching feedback should therefore not automatically be interpreted as indicators of limited long-term potential. In many cases, such behavioral changes may instead represent transient stress responses requiring supportive intervention, individualized communication, and temporary training adjustment.

Psychological and behavioral assessment within real-world academy environments also remains inherently subjective. Coach observations, communication patterns, training behaviors, and perceived attitudes may provide valuable practical information, but these interpretations are also vulnerable to observer bias, interpersonal dynamics, and contextual expectations. Whenever feasible, repeated observations obtained across different training and competition contexts, together with structured communication between coaching, medical, performance, and support staff, may improve the consistency and contextual interpretation of psychological assessment. Practical wellness questionnaires or simplified psychometric monitoring tools may additionally provide useful complementary information when feasible within academy environments, although their interpretation should always acknowledge their subjective nature and complement rather than replace routine coaching observations.

From an applied perspective, youth football environments should prioritize supportive systems capable of facilitating both football-specific skill acquisition and long-term psychosocial development. Constructive coach-athlete communication, individualized feedback, learning-oriented training environments, emotional support, and realistic developmental expectations may positively influence adaptation quality during adolescence. Psychological and perceptual-cognitive evaluation should therefore primarily support practical coaching decisions, athlete support, and developmentally appropriate management within academy settings, as illustrated in Box 1.

Box 1 **Contextualizing Behavioral Fluctuations: Applied Intervention Strategies.**

  A central principle of athlete management in youth football is recognizing that similar behavioral or cognitive changes may require different practical interventions depending on the underlying developmental context.

  For example, two U15 athletes may present a sudden decline in training engagement, emotional stability, and on-pitch decision-making quality over a three-week period. A simplified interpretation might classify both athletes as “unmotivated” or “lacking tactical focus.” However, repeated reassessment may reveal substantially different underlying contributors.

  Athlete A

  Behavioral changes coincide with an intense academic examination period, consistently reduced sleep quality, and symptoms compatible with accumulated mental fatigue.

  Applied Intervention: Temporary reduction in training volume and cognitive demands, simplified tactical responsibilities during training, increased recovery emphasis, and appropriate management of academic and psychosocial stressors to reduce cumulative physical and psychological stress.

  Athlete B

  Behavioral decline coincides with Peak Height Velocity (PHV), where transient coordination disturbances (“adolescent awkwardness”) have resulted in repeated technical errors during competitive situations, contributing to frustration and temporary reductions in confidence.

  Applied Intervention: Reassurance from coaching staff, temporary transition from outcome-based to process-oriented feedback, mobility support, and maturation-appropriate movement interventions recognizing the behavior as a secondary consequence of biological growth rather than reduced motivation.

This applied framework highlights the importance of individualized communication, longitudinal monitoring, and developmentally appropriate intervention during adolescence. Importantly, similar behavioral presentations may reflect fundamentally different underlying mechanisms; consequently, effective athlete management depends on contextual interpretation rather than isolated behavioral observations.

## 6. Interaction Between Talent and Physical Constraints

### Performance Expression Beyond Isolated Measurements

Technical, tactical, physical, and maturational characteristics rarely influence football performance independently. Instead, performance expression emerges from the interaction between an athlete’s football-specific abilities and the physical constraints imposed by growth, biological maturation, training status, fatigue, and environmental demands [23,59,60]. Understanding these interactions is essential for distinguishing temporary limitations from true developmental potential and for guiding individualized athlete-management decisions within youth football academies.

In practice, these interactions frequently manifest as compensation mechanisms within academy environments. Rather than reflecting fixed strengths or weaknesses, compensation may change throughout adolescence as biological maturation, training adaptations, and football-specific expertise evolve. Superior game intelligence, spatial anticipation, and technical efficiency may partially compensate for temporary limitations in acceleration or maximal force production [2,4,22,23]. During adolescence, where physical development remains highly variable, this compensation may become particularly important for maintaining competitive effectiveness against physically mature opponents.

For example, a technically gifted central midfielder may consistently identify progressive passing options and demonstrate advanced positional awareness yet still struggle to execute actions effectively during high-tempo transitions due to insufficient acceleration capacity or reduced movement efficiency under fatigue. Such examples illustrate that football performance depends not only on tactical perception or physical output independently, but on the interaction between them during competitive situations.

Conversely, physical characteristics may also function as practical performance limiters on technical and tactical expression. Rather than determining football potential, physical capacity influences the extent to which football-specific abilities can be consistently expressed under the temporal and physiological demands of competition. Insufficient physical capacity may limit the consistent expression of football-specific abilities during demanding match situations, even when technical, tactical, and perceptual skills are well developed. During high-intensity match situations, an athlete may possess advanced decision-making capabilities, yet fatigue accumulation, insufficient force production, or reduced movement efficiency may prevent the athlete from reaching the required spatial positions quickly enough to execute those decisions effectively [1,4,61]. Accordingly, athlete management should aim not only to develop football-specific expertise but also to ensure that physical capacities are sufficient to support its consistent expression during competition.

These interactions may additionally vary according to positional role, tactical system, and maturation status. The movement strategies and physical requirements of wide attacking players differ substantially from those of central midfielders, central defenders, or forwards, resulting in distinct technical, tactical, and physiological priorities across positions [62]. Similarly, the influence of physical constraints may change throughout adolescence as biological maturation alters the relative contribution of physical and football-specific characteristics to performance expression [8,39].

Rather than searching for idealized physical profiles, applied athlete-management processes should aim to identify which specific limitations currently restrict football-specific performance. Once a limiting factor is identified -whether related to acceleration mechanics, movement efficiency, mobility, force production, or fatigue tolerance- practitioners may implement targeted interventions designed to reduce these constraints while simultaneously supporting football-specific development. Within academy environments, multidimensional athlete evaluation should therefore primarily support individualized training adjustment, developmental planning, and sustainable athlete progression. Importantly, the practical value of this multidimensional approach depends not on any single assessment outcome, but on the repeated integration of football-specific, physical, maturational, and psychological information to guide individualized athlete-management decisions over time.

## 7. Recovery, Load Management, and Sustainable Athlete Development

### Supporting High-Level Training Without Burnout

Recovery represents a fundamental component of athlete progression extending far beyond simple post-exercise regeneration. Session-RPE is commonly calculated as training duration multiplied by perceived exertion score and may provide a practical estimate of internal training load when collected consistently approximately 30 min after session completion [63]. Within demanding academy environments, an athlete’s ability to tolerate repeated training exposure, adapt positively to workloads, and maintain long-term progression is strongly influenced by the interaction between training stimulus, biological maturation, recovery quality, and individual stress tolerance [3,64,65]. Recovery management should therefore be integrated directly into everyday athlete-management processes during adolescent development.

This interaction becomes particularly important during periods of rapid growth. As discussed in previous sections, rapid skeletal development and temporary coordination disturbances may substantially alter movement mechanics and neuromuscular efficiency. From a load-management perspective, reduced mechanical efficiency may significantly increase the physiological cost of training. A standard session that previously elicited a moderate internal load may suddenly provoke excessive fatigue responses in an athlete undergoing Peak Height Velocity (PHV) [66]. If practitioners fail to reassess training demands during these phases, temporary developmental constraints may contribute to inappropriate loading strategies and increase the risk of lower-extremity injury during adolescence [47].

Youth athletes additionally demonstrate considerable variability in their responses to identical training stimuli. Maturation status, sleep quality, nutritional habits, academic burden, psychosocial stress, recovery capacity, and cumulative competitive exposure may all influence adaptation and fatigue responses between athletes of similar chronological age [67]. Consequently, similar monitoring outcomes may require different practical responses. For example, two players may report unusually elevated session-rating of perceived exertion (sRPE) values following a routine tactical training session. In one athlete, this increased internal load may primarily reflect unresolved neuromuscular fatigue from previous competitive exposure, requiring temporary recovery emphasis. In another athlete, the same sRPE response may relate more closely to mental fatigue, academic stress, or insufficient sleep quality, requiring schedule adjustment, psychological support, or temporary cognitive deloading strategies.

Physical and mental recovery should be considered complementary but distinct components of athlete management. Whereas physical recovery primarily aims to restore neuromuscular function, tissue homeostasis, and physiological readiness, mental recovery focuses on reducing accumulated cognitive effort, emotional stress, attentional demands, and decision-making fatigue. Recent evidence indicates that mental fatigue may develop independently of physical fatigue and may negatively influence technical execution, tactical behaviour, reaction time, vigilance, and perceptual-cognitive performance in football [54,55,56]. Consequently, recovery strategies should be individualized according to the predominant source of fatigue rather than assuming that all fatigue responses require identical interventions.

Within competitive academy structures, excessive training exposure, congested schedules, persistent performance pressure, and early specialization may further increase the risk of maladaptation, psychological fatigue, reduced motivation, and burnout during sensitive developmental phases [68]. Sustainable athlete progression should therefore depend not simply on increasing training volume, but on the practitioner’s ability to adjust demands according to the athlete’s recovery status, maturational stage, and stress tolerance.

From an applied perspective, effective recovery management depends not only on organizational resources, but also on the athlete’s understanding and long-term adoption of recovery-related behaviors. Physiotherapy support, hydrotherapy, regeneration sessions, individualized mobility work, massage therapy, and recovery-oriented scheduling may all contribute positively within academy environments. However, the effectiveness of these interventions may be substantially reduced when athletes lack sufficient awareness regarding sleep hygiene, hydration, nutrition, fatigue recognition, or adherence to recovery recommendations. Athlete education should therefore represent an important component of long-term developmental support. Similarly, coach education and interdisciplinary communication have increasingly been recognized as critical elements of effective monitoring implementation, particularly within environments where coaches must simultaneously manage performance, recovery, and developmental decision-making [4,69].

In many real-world academy environments, sophisticated monitoring technologies may not always be financially feasible or practically available. Nevertheless, validated low-cost approaches such as session-rating of perceived exertion (sRPE), wellness questionnaires, observational monitoring, coach-athlete communication, and individualized training feedback may still provide meaningful information regarding fatigue status, recovery quality, and training tolerance [70,71,72]. Implemented through smartphone-based questionnaires, simplified digital forms (e.g., Google Forms), or routine communication workflows, these approaches require minimal technological infrastructure. Recent advances in wearable sensor technology may further complement these practical approaches when resources permit, although technological sophistication should not replace consistent longitudinal monitoring and contextual interpretation of athlete responses [33]. In many academy settings, the primary challenge is not data collection itself, but the consistent interpretation and communication of monitoring information across coaching staff.

Load management should therefore function as a practical process supporting training adjustment, recovery planning, injury-risk reduction, and long-term athlete availability within youth football environments. Ultimately, sustainable athlete development depends not only on monitoring training load itself, but on the continuous integration of recovery status, biological maturation, football-specific performance, psychological well-being, and individual contextual factors to guide informed athlete-management decisions over time.

## 8. Practical Applications in Real-World Academy Settings

### Applied Multidimensional Assessment Without Advanced Laboratory Infrastructure

**Figure 1:**
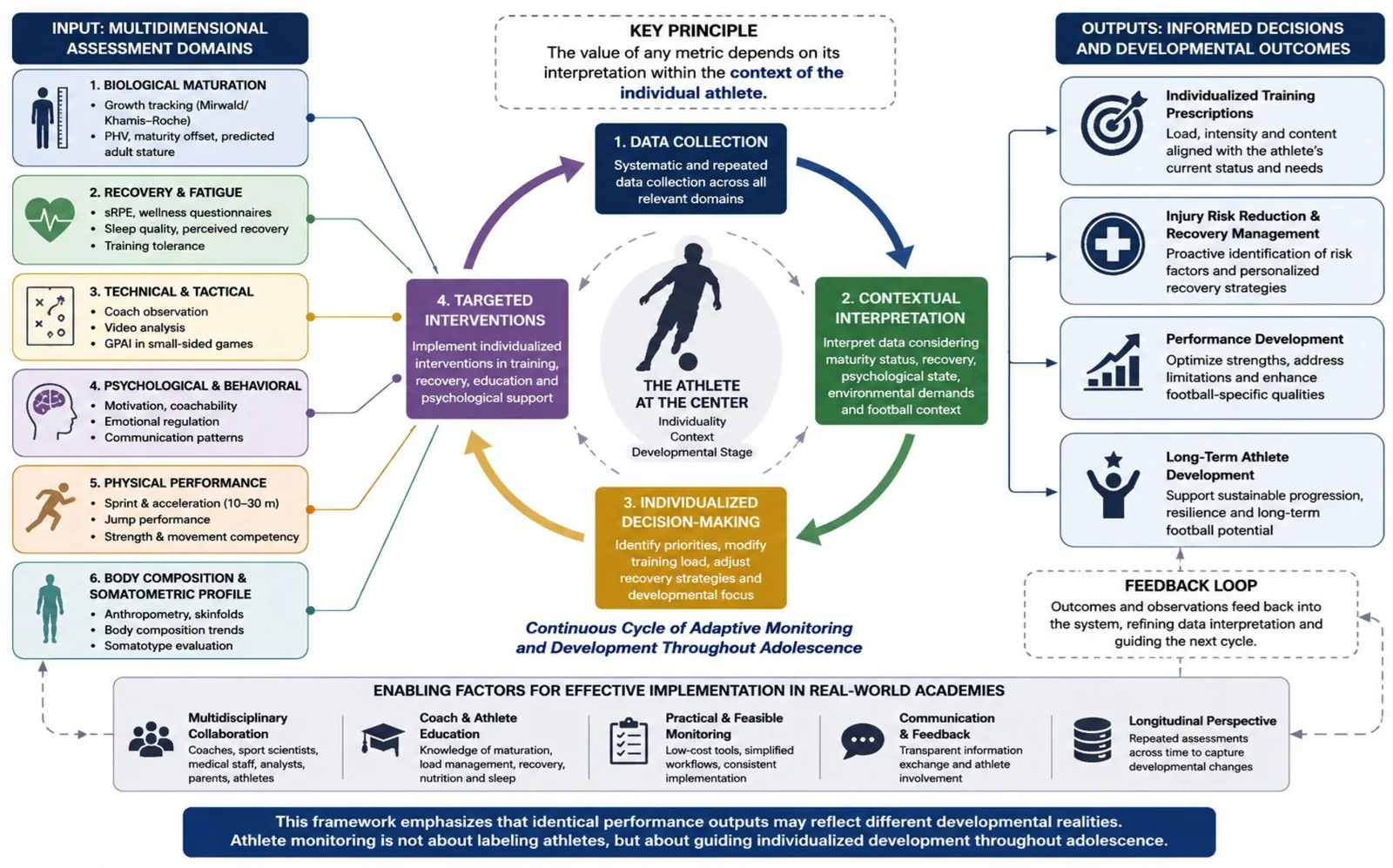
Adaptive Athlete Management Framework for Youth Football Academies.

In real-world youth academies, athlete evaluation frequently occurs within environments constrained by limited financial resources, restricted staffing, and high coach workloads. Although advanced biomechanical laboratories and high-resolution tracking systems may provide valuable physiological information, they are not prerequisites for effective athlete management. The primary objective is not maximal technological sophistication, but the collection of sufficiently reliable information capable of supporting consistent longitudinal monitoring. As illustrated in Figure 1 and summarized in Table 1 and Table 2, combining field-based assessment, maturation estimation, wellness monitoring, and structured observational analysis may create an operationally feasible monitoring process within academy settings.

The framework illustrates how multidimensional assessment, contextual interpretation, individualized decision-making, and targeted interventions interact within a continuous cycle of athlete management in youth football. The model emphasizes longitudinal monitoring and developmentally informed support throughout adolescence.

While Table 1 summarizes the principal assessment domains, monitoring tools, and their interpretation, Table 2 provides an example of how these domains may be implemented in routine academy practice through a flexible multidimensional monitoring schedule. The proposed framework (Figure 1) illustrates how multidimensional assessment, contextual interpretation, individualized decision-making, and targeted interventions interact within a continuous cycle of athlete management in youth football. As a conceptual framework, it is intended to support practical decision-making and should be considered a guide for applied practice pending future longitudinal validation in academy environments.

Several approaches may substantially improve monitoring quality without requiring advanced infrastructure. Non-invasive methods such as the Mirwald equations or the Khamis–Roche method may provide useful biological context, allowing practitioners to consider maturational status during physical-performance evaluation [48,49]. For fatigue and load management, session-rating of perceived exertion (sRPE) and digital wellness questionnaires offer accessible estimations of internal load and recovery status [70,71]. When implemented consistently through simple digital workflows (e.g., smartphone applications or customized digital forms), these tools may generate useful longitudinal information with minimal financial investment. Where available, wearable sensor technologies may provide complementary information to field-based monitoring; however, their value depends primarily on appropriate interpretation, methodological consistency, and integration into longitudinal athlete management rather than on technological sophistication alone [4,33].

When assessing physical performance, methodological consistency remains essential. Manual stopwatch timing introduces considerable measurement error over short sprint distances, often exceeding true biological performance fluctuations [28]. Basic electronic timing gates or validated smartphone applications for neuromuscular profiling (e.g., MyJump) provide accessible alternatives demonstrating acceptable agreement with laboratory standards [29,31]. As summarized in Table 1 and Table 2, the usefulness of these tools depends less on technological complexity and more on consistent implementation, repeated monitoring, and effective communication between staff members.

**Figure 2:**
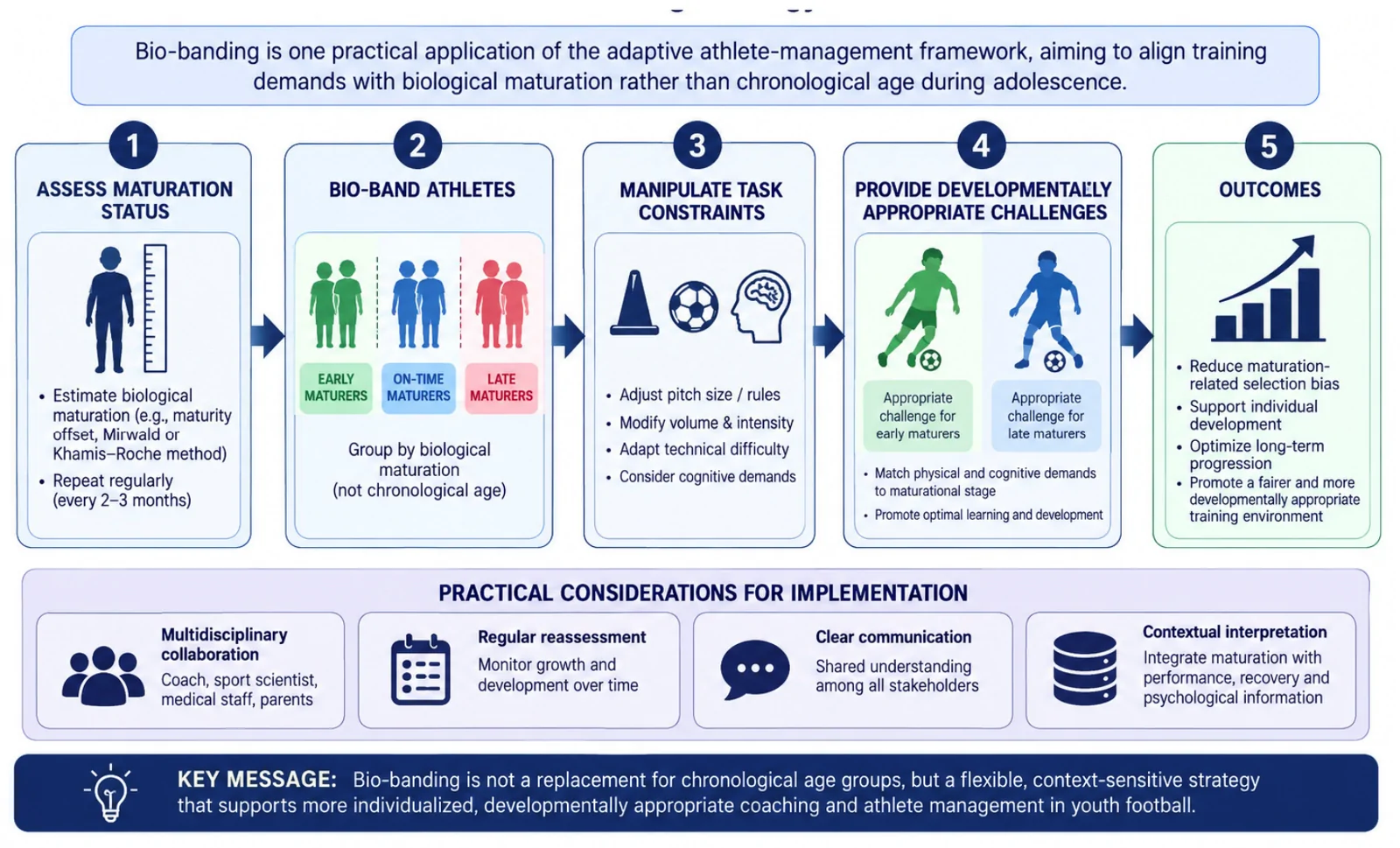
Intra-Club Bio-Banding: Translating Maturational Information into Individualized Coaching Strategies.

Objective monitoring data only possesses value when it directly informs coaching interventions. Numerical outputs cannot replace structured coach observation, match analysis, and behavioral assessment. As illustrated in Figure 2, and further exemplified in Box 1, similar monitoring outcomes may require different practical responses depending on the athlete’s maturational status, psychological condition, recovery profile, and current training demands. One potential practical application of this multidimensional approach is intra-club bio-banding. By periodically grouping athletes according to biological maturation rather than chronological age, academies may modify physical exposure, tactical demands, and developmental challenges according to the athlete’s current stage of development. Bio-banding has been proposed as one strategy to help reduce maturation-related selection bias while facilitating developmentally appropriate physical, technical, tactical, and cognitive challenges; however, evidence supporting its long-term effects on athlete development remains limited [43,44].

The figure illustrates one practical application of the Adaptive Athlete Management Framework presented in Figure 1. It demonstrates how biological maturation can be incorporated into athlete grouping, task manipulation, and individualized coaching to provide developmentally appropriate training challenges and support long-term athlete development.

The effectiveness of athlete-monitoring systems depends heavily on multidisciplinary communication. Even sophisticated monitoring approaches may become ineffective if assessment results are poorly interpreted, inconsistently communicated, or disconnected from everyday training decisions. Conversely, simple low-cost monitoring systems may still provide substantial applied value when embedded within consistent communication workflows between coaching, medical, and performance staff [4]. Accordingly, the implementation schedule presented in Table 2 should be interpreted as an indicative, flexible example rather than a prescriptive monitoring protocol. Monitoring frequency should always be adapted to athlete age, biological maturation, competitive schedule, available resources, and the specific objectives of each academy.

Within real-world academy environments, effective athlete monitoring should prioritize operational feasibility and coaching applicability rather than technological complexity alone. Ultimately, the practical value of any monitoring system depends less on technological sophistication than on the consistent collection of meaningful information, contextual interpretation, interdisciplinary communication, and its translation into individualized coaching and athlete-management decisions.

## 9. Limitations and Interpretation Challenges

### Contextual and Structural Constraints in Athlete Evaluation

Despite the practical value of multidimensional athlete evaluation in youth football, several important methodological and applied limitations should be acknowledged. Football performance and long-term athlete progression remain highly complex processes influenced by technical, tactical, physical, psychological, maturational, and environmental factors. Consequently, single assessments should not be viewed as definitive indicators of present or future football potential, particularly during adolescence where developmental variability remains substantial [8,9,23]. The framework proposed in the present review is intended to support athlete-management decisions within academy environments rather than function as a predictive talent-identification model. As a conceptual framework, it should be viewed as a decision-support approach requiring prospective longitudinal evaluation across diverse academy settings before its practical effectiveness can be established.

Biological maturation also represents an important interpretative limitation. Because maturational status substantially influences physical performance during adolescence, findings from individual assessments should always be interpreted within the athlete’s developmental context rather than chronological age alone [8,40,43].

Field-based assessments are also subject to methodological variability. Consequently, performance outcomes should be interpreted within the context of standardized testing procedures and consistent longitudinal monitoring rather than as isolated measurements [4,28,31,35,53].

Additional complexity arises from the subjective nature of several football-specific assessment domains. Technical execution, tactical understanding, decision-making, positional intelligence, and psychological characteristics frequently require observational interpretation by coaches or practitioners, introducing the potential for observer bias, inconsistency, and contextual variability. Even when structured observational frameworks are utilized, football-specific evaluation remains partially dependent on coaching philosophy, competitive demands, and evaluator experience [73,74]. Structured communication between coaches, sports scientists, physiotherapists, and performance staff may help reduce interpretative inconsistency and improve decision-making within academy environments.

Another important limitation relates to the potential over-standardization of athlete profiling models. Although physical and performance assessments may provide useful developmental information, football performance itself remains dynamic, interactive, and highly dependent on positional role, tactical system, playing style, and competitive demands. Attempts to define universal or idealized athlete profiles may therefore oversimplify the complexity of football progression and underestimate the value of diverse developmental pathways. In practice, athletes may achieve effective football performance through different combinations of technical, tactical, psychological, and physical strengths.

Resource-related limitations should also be acknowledged within many academy environments. Restricted staffing availability, limited technological infrastructure, financial constraints, inconsistent longitudinal monitoring, and reduced time availability may substantially influence the quality and continuity of athlete-evaluation processes. Although recent advances in wearable sensor technologies and digital monitoring platforms have increased the accessibility of athlete monitoring, successful implementation continues to depend primarily on organizational resources, staff expertise, and consistent communication rather than technology alone [33]. Consequently, even theoretically valuable monitoring systems may prove difficult to implement consistently within applied football settings.

Athlete progression should be interpreted longitudinally, recognizing that temporary fluctuations during adolescence do not necessarily reflect long-term developmental potential [9,47].

Ethical considerations should likewise be acknowledged when implementing athlete-assessment systems within youth football environments. Excessive emphasis on early selection, rigid performance categorization, or premature exclusion based primarily on temporary physical advantages may negatively influence athlete confidence, motivation, psychosocial well-being, and long-term participation [72]. Athlete-management approaches should therefore support equitable opportunity, sustainable participation, and developmentally appropriate progression.

Although the proposed framework has been developed from extensive practical experience within a professional academy environment, its broader effectiveness has not yet been evaluated prospectively across different academy settings. Furthermore, the present review does not extensively examine the potential influence of parental involvement, family expectations, and coach–parent interactions on athlete development, despite their recognized impact on psychological adaptation, recovery behaviors, communication dynamics, and long-term participation during adolescence. Future prospective studies should evaluate the implementation, feasibility, reliability, and practical effectiveness of the proposed framework across different academy structures, competitive standards, and developmental contexts.

Overall, multidimensional athlete monitoring should be viewed as a decision-support process that complements expert coaching judgement, individualized athlete support, and the contextual interpretation of football performance rather than replacing them.

## 10. Future Directions

### Toward Continuous and Individualized Athlete Development Models

Future approaches to athlete development in youth football should increasingly prioritize longitudinal monitoring, contextual interpretation, and athlete-specific management rather than isolated cross-sectional testing procedures. Repeated assessment during adolescence may assist practitioners in identifying emerging physical limitations, transient coordination disturbances, changing recovery responses, and adaptation patterns more effectively than isolated one-time evaluations. Future longitudinal research should also investigate how multidimensional monitoring influences coaching decision-making and individualized athlete management rather than focusing exclusively on isolated performance outcomes.

Future research should focus on the prospective longitudinal validation of practical athlete-management models within applied academy environments. In particular, future studies should evaluate the feasibility, implementation, reliability, and practical effectiveness of the Adaptive Athlete Management Framework proposed in this review across different academy structures, competitive standards, and developmental settings. Additional evidence is required regarding how multidimensional monitoring strategies influence injury risk management, training tolerance, recovery management, player availability, and football progression during adolescent development.

Further integration between football-specific observation and physical-performance assessment also represents an important future direction. Although sprint performance, jump capacity, body composition, and force-production characteristics contribute useful developmental information, football performance remains strongly influenced by technical execution, tactical understanding, perceptual-cognitive abilities, and decision-making quality during competitive play. Combining objective monitoring data with structured coach observation, match analysis, and athlete feedback may therefore improve coaching decisions and developmental planning within academy settings. Future research should additionally investigate how objective monitoring information can be integrated with structured observational assessment to improve contextual interpretation rather than replace expert coaching judgement.

As wearable sensors, automated video analysis, and AI-assisted decision-support systems continue to expand, future technological developments should prioritize practical usability, interpretability, and operational feasibility rather than technological complexity alone. Advanced analytical systems may process large datasets efficiently; however, coaching expertise remains essential for integrating objective information into individualized training adjustment and athlete support. Similarly, future technological developments should emphasize transparent, explainable, and clinically interpretable outputs that facilitate communication between coaching, medical, and performance staff rather than generating increasingly complex data alone.

Importantly, future athlete-development strategies must remain applicable within real-world academy environments rather than relying exclusively on highly resourced elite infrastructures. Low-cost but methodologically consistent monitoring approaches may still provide meaningful developmental information when implemented systematically over time.

The effectiveness of future athlete-management systems will additionally depend heavily on multidisciplinary communication and educational support. Coaches, sports scientists, physiotherapists, medical staff, strength and conditioning professionals, athletes, and parents may all contribute meaningfully to the implementation of monitoring information. Improving education regarding maturation, recovery management, fatigue monitoring, sleep hygiene, nutrition, and long-term athlete development may further strengthen decision-making and athlete support during adolescence. Future implementation research should also examine educational strategies capable of improving practitioners’ interpretation of multidimensional monitoring information and its translation into everyday coaching practice.

## 11. Conclusions

This review proposes a multidimensional framework to support athlete evaluation and management in youth football by integrating football-specific, physical, maturational, psychological, and contextual information within a longitudinal decision-making process. Rather than relying on isolated test outcomes, the framework emphasizes individualized and context-sensitive interpretation to guide athlete development and informed decision-making throughout adolescence.

The perspectives presented throughout this review support the need for developmentally informed athlete-management approaches within youth football academies. Physical testing, maturation monitoring, somatometric assessment, recovery tracking, psychological observation, and football-specific evaluation may all contribute valuable developmental information; however, their practical value depends on how effectively they are integrated, interpreted, and translated into individualized coaching decisions within the athlete’s developmental context.

Importantly, athletes may achieve competitive effectiveness through different combinations of technical, tactical, psychological, and physical strengths. Accordingly, athlete monitoring should not seek to identify a single developmental profile but rather support individualized progression through context-sensitive interpretation and longitudinal observation.

Within real-world academy environments, effective athlete management does not necessarily require highly sophisticated technologies or extensive laboratory infrastructure. Methodologically consistent and operationally feasible monitoring approaches, combined with structured observation, multidisciplinary communication, athlete education, and contextual interpretation of monitoring information, may provide meaningful support for sustainable progression and athlete availability over time.

Overall, athlete monitoring in youth football should function primarily as a practical support process designed to guide individualized development, training adjustment, recovery management, and football progression within adolescent athlete populations. When implemented within a developmentally informed, multidisciplinary, and athlete-centred framework, multidimensional monitoring has the potential to enhance coaching decision-making while supporting sustainable long-term athlete development rather than simply generating additional performance data.

## Figures and Tables

**Table 1:** Practical Multidimensional Assessment and Monitoring Approaches in Real-World Youth Football Academies.

Assessment Domain	Practical Low-Cost/Valid Approach	Practical Information Provided	Measurement Error Risk & Implementation Considerations	Interaction with Football-Specific Talent and Development	Potential Adaptive Interpretation
**Biological Maturation**	Longitudinal growth tracking using Mirwald equations or Khamis–Roche method	Somatic maturity offset, predicted adult stature, identification of Peak Height Velocity (PHV)	Requires standardized anthropometric measurements (height, sitting height, body mass), assessor consistency, and repeated monitoring intervals; prediction equations should be interpreted cautiously during adolescence	Helps contextualize temporary physical advantages and supports contextual reinterpretation of physical performance during developmental transitions, reducing the risk of premature exclusion of late-maturing players with high technical or tactical potential	Similar physical outputs may reflect different maturational stages rather than equivalent developmental status
**Recovery & Fatigue Monitoring**	Session-RPE (sRPE), wellness questionnaires, individualized athlete communication, smartphone- or Google Forms–based monitoring	Internal training load, subjective recovery status, fatigue perception, sleep quality, training tolerance	Dependent on athlete honesty, compliance, and consistent implementation; interpretation may be influenced by emotional, academic, or psychosocial stressors	Supports individualized load adjustment and continuous reinterpretation of training tolerance across developmental phases and periods of increased fatigue	Elevated fatigue scores may reflect cumulative training load, sleep disturbance, academic stress, psychological fatigue, or reduced recovery capacity
**Technical & Tactical Evaluation**	Structured coach observation, video-assisted match analysis, GPAI-based evaluation during small-sided games	Decision making, positioning, game understanding, technical execution, tactical adaptability under pressure	Requires predefined observational criteria, observer consistency, and longitudinal evaluation to reduce subjectivity	Represents the central component of football-specific evaluation and contextualizes physical and developmental performance data within real-game behavior	Similar technical outcomes may emerge through different developmental pathways depending on maturation, tactical understanding, or physical compensation strategies
**Psychological & Behavioral Characteristics**	Behavioral observation, communication patterns, coach feedback, longitudinal athlete interaction	Motivation, adaptability, emotional regulation, coachability, competitive responsiveness	Subjective interpretation requires structured communication between coaching and performance staff members	Influences adaptation, response to feedback, recovery adherence, and individualized developmental progression over time	Reduced engagement or inconsistent behavior may reflect psychological fatigue, external stressors, confidence fluctuations, or maladaptive training responses
**Sprint & Acceleration Assessment**	Electronic timing gates with standardized sprint initiation procedures and split-time analysis over 10–30 m	Acceleration ability, transition speed, sprint structure, repeated high-intensity movement capacity	Manual stopwatch timing may introduce substantial systematic error in short-distance sprint testing; environmental conditions and familiarization procedures should be standardized	High acceleration capacity may support repeated high-intensity football actions and transition efficiency, although technical and tactical qualities may partially compensate for physical limitations	Similar sprint outputs may reflect different maturational, neuromuscular, fatigue-related, or movement-coordination states and therefore require contextual longitudinal interpretation
**Jump Performance**	Validated smartphone applications (e.g., MyJump) or contact jump mats	Lower-limb power, explosiveness, neuromuscular readiness, training adaptation trends	Technical execution consistency, familiarization, and movement-strategy variability may influence reliability	Provides supportive information regarding neuromuscular performance and adaptation throughout development	Reduced jump performance may reflect neuromuscular fatigue, altered movement strategy, insufficient force production, or maturation-related coordination changes
**Strength Assessment**	Field-based strength testing, resistance-training monitoring, movement competency evaluation	Force-production characteristics, physical robustness, deceleration control, movement stability	Requires standardized testing procedures, technical supervision, and consistent movement execution	May support physical resilience, repeated high-intensity actions, fatigue tolerance, and movement efficiency during football competition	Strength deficits may function as rate-limiting constraints affecting acceleration efficiency, deceleration control, or injury resilience
**Body Composition & Somatometric Profile**	Body mass tracking, skinfold assessment, somatotype evaluation, longitudinal anthropometric monitoring	Morphological characteristics, body-composition trends, developmental profile	Requires assessor consistency and cautious interpretation during periods of rapid growth and maturation	May influence movement efficiency, movement strategies, and physical expression within specific football contexts while requiring contextual developmental interpretation	Temporary morphological changes during growth may alter coordination, movement efficiency, or physical expression without indicating reduced long-term potential
**Practical Academy Constraints**	Multidisciplinary communication, simplified monitoring workflows, selective use of low-cost technologies	Feasible long-term developmental support within real-world academy environments	Time limitations, staffing availability, coach workload, and inconsistent implementation may affect monitoring quality	Supports sustainable athlete monitoring, contextual interpretation, and individualized developmental decision making within practical academy settings	Simplified monitoring systems may still provide meaningful developmental insight when interpreted longitudinally within coherent adaptive-management frameworks

**Table 2:** Suggested Practical Implementation of Multidimensional Athlete Monitoring in Youth Football Academies.

Assessment Domain	Suggested Frequency *	Primary Purpose	Personnel Primarily Involved	Minimum Practical Implementation
**Wellness monitoring**	Daily or before each training session	Assess perceived recovery, fatigue, soreness, sleep quality and readiness	Athlete, coach, sport scientist	Brief wellness questionnaire (paper or digital)
**Internal training load (session-RPE)**	After every training session and match	Quantify individual training load and monitor cumulative stress	Athlete, coach, sport scientist	Standardized session-RPE collection
**External load (where available)**	Every training session and match	Monitor locomotor demands and training exposure	Sport scientist, S&C coach	GPS or alternative field-based monitoring
**Physical performance assessment (e.g., sprint, jump, strength)**	Periodically (e.g., after defined training blocks or every 4–8 weeks, depending on objectives)	Monitor neuromuscular performance and long-term development	S&C coach, sport scientist	Standardized field-based performance testing
**Growth, anthropometry and biological maturation**	Every 2–3 months during adolescence or periods of rapid growth	Monitor growth-related changes and support maturation-informed interpretation	Medical staff, S&C coach	Height, body mass and maturity estimation
**Psychological and behavioural monitoring**	Ongoing, with structured review when indicated	Identify wellbeing concerns, motivation, stress and behavioural changes	Coach, psychologist (where available), multidisciplinary staff	Structured observation and athlete communication
**Technical–tactical evaluation**	Continuous throughout training and competition	Assess football-specific performance and decision-making	Coaching staff	Match and training observation using standardized criteria where feasible
**Integrated multidisciplinary athlete review**	Periodically (e.g., every 6–8 weeks or following major training phases)	Integrate information from multiple domains to support individualized athlete-management decisions	Multidisciplinary team	Multidomain review of monitoring data and athlete profile

* *Suggested frequencies represent indicative practice recommendations informed by the current literature and applied experience rather than prescriptive guidelines. The optimal monitoring schedule should be individualized according to athlete age, biological maturation, competitive schedule, available resources, and the objectives of each academy.*

## Data Availability

No new data were created or analyzed in this study. Data sharing is not applicable to this article.
